# Photothermocapillary Method for the Nondestructive Testing of Solid Materials and Thin Coatings

**DOI:** 10.3390/s21196671

**Published:** 2021-10-07

**Authors:** Aleksandr Zykov, Vladimir Vavilov, Marina Kuimova

**Affiliations:** 1 Research Laboratory of Photonics and Microfluidics, Tyumen State University, 625003 Tyumen, Russia; aleksandr.zykov@inbox.ru; 2 Department of Vocational Guidance and Pre-University Training, Tyumen Industrial University, 625000 Tyumen, Russia; 3 Engineering Institute of Nondestructive Testing, National Research Tomsk Polytechnic University, 634050 Tomsk, Russia; kuimova@tpu.ru

**Keywords:** photothermocapillary effect, nondestructive testing, defect, varnish-paint coating, delamination, surface tension

## Abstract

The photothermocapillary (PTC) effect is a deformation of the free surface of a thin liquid layer on a solid material that is caused by the dependence of the coefficient of surface tension on temperature. The PTC effect is highly sensitive to variations in the thermal conductivity of solids, and this is the basis for PTC techniques in the non-destructive testing of solid non-porous materials. These techniques analyze thermal conductivity and detect subsurface defects, evaluate the thickness of thin varnish-and-paint coatings (VPC), and detect air-filled voids between coatings and metal substrates. In this study, the PTC effect was excited by a “pumped” Helium-Neon laser, which provided the monochromatic light source that is required to produce optical interference patterns. The light of a small-diameter laser beam was reflected from a liquid surface, which was contoured by liquid capillary action and variations in the surface tension. A typical contour produces an interference pattern of concentric rings with a bright and wide outer ring. The minimal or maximal diameter of this pattern was designated as the PTC response. The PTC technique was evaluated to monitor the thickness of VPCs on thermally conductive solid materials. The same PTC technique has been used to measure the thickness of air-filled delaminations between a metal substrate and a coating.

## 1. Introduction

Microfluidics involves the manipulation of small volumes of liquids. The use of microfluidics in engineering, chemistry, and biology, including thermocapillary-based sensors and devices, has increased in recent years and has been summarized by Karbalaei et al. [1]. A review of digital microfluidic devices was presented by Shiyu Chen et al. [2]. Jasińska and Malecha described the proposed microfluidic modules with integrated microwave components [3].

Kalliadasis et al. considered a two-dimensional model of the motion of a liquid film to predict local film heights and temperature distributions as a function of time [4]. Zahurul Islam and Ying Yin Tsui developed a quasi-3D model to simulate the flow in planar microfluidic devices [5]. Wenjun Liu et al. [6] and Mohammadbar et al. [7] used the numerical modeling of the evaporation from a liquid layer by considering the phenomena of heat and mass transfer. The kinetic aspects of the Marangoni phenomenon in viscous polymer solutions were investigated by Ye Tian et al. [8]. One example of a new application of microfluidics is the microrobotic platform proposed by the authors of [9]. Flow-focusing microfluidic devices were used in dielectrophoretic applications [10].

Several investigations have been performed in areas related to the research conducted in this study. The mechanism of heat exchange was investigated in an analysis of the instability caused by the thermocapillary effect to show that the mass evaporation rate decreases with increasing thickness of an evaporating layer, with a maximum value appearing at a thickness of 5 mm [11].

Thermocapillary convection in the presence of a surfactant film was studied to illustrate the importance of temperature profiles, which provided important information on the tangential stresses and the degree of compression of the surfactant film [12]. Jing Zhang et al. suggested a material-independent method for manipulating in-fiber particles based on laser-induced thermocapillary convection [13]. The thermocapillary phenomena of phase-change materials were studied by Salgado Sánchez et al. [14]. Terrazas et al. demonstrated that heating a fluid from above results in independence from hydrodynamic instability to initiate fluid flow [15]. Pin-Chuan Chen et al. developed an automated optical inspection system intended to rapidly and precisely measure the dimensions of microchannels embedded in a transparent polymeric substrate [16].

The photothermocapillary (PTC) effect is based on the deformation of the free (top) surface of a thin liquid layer (<1 mm) on the surface of a solid material. This deformation is caused by the dependence of the coefficient of liquid surface tension $\sigma_{0}$ on temperature T: $\sigma =\sigma_{0}-\left|d\sigma /dT\right|\left(T-T_{0}\right)$, where $T_{0}$ is the ambient temperature, and $\sigma^{\prime }=d\sigma /dT$ is the thermal coefficient of surface tension (derivative of the surface tension with respect to the temperature). Another report states that the PTC effect is a thermocapillary (TC) flow, since fluid flow can take place at a very low velocity in the absence of deformation [1]. In general, a temperature change, $\Delta T=T-T_{0}$, on the surface of a liquid can be induced using various heat sources, including focused electromagnetic radiation of any wavelength (ultraviolet, visible, or infrared) [12,17,18,19,20,21,22,23]. In two limiting cases, PTC flow takes place in a liquid, which strongly absorbs the electromagnetic radiation at its surface, and/or in a liquid, which is either transparent or weakly absorbs the radiation of a pumped laser or a halogen lamp [13]. In the first case, the substrate can have any absorption coefficient $a_{s}$, while in the second case, the substrate should absorb a maximum amount of the pumped laser radiation, i.e., $a_{s}$ = 1. The morphology of PTC flows, particularly Marangoni flows around micro bubbles, can be effectively studied using the laser-induced photothermal technique [24]. The general analysis of photothermal phenomena was reviewed by Terazima et al., who also presented the related quantities, terminology, and symbols of terms [25]. Vargas reviewed the use of photothermal methods for determining material thermal properties, such as the thermal diffusivity and thermal effusivity [26].

The analysis of numerous investigations on the thermocapillary effect shows the influence of the thermal conductivity of the substrate on the characteristics of thermocapillary flow has not been sufficiently addressed. In this study, we define the relationship between the thermal conductivity of the substrate and the diameter of PTC optical patterns, and suggest a novel technique to determine the thickness of the varnish-and-paint coating (VPC). In addition, a non-destructive testing (NDT) method to detect delaminations between a VPC and a substrate, and discontinuities in solids is proposed.

## 2. Methods and Materials

### 2.1. Theory of PTC Effect

**Figure 1:**
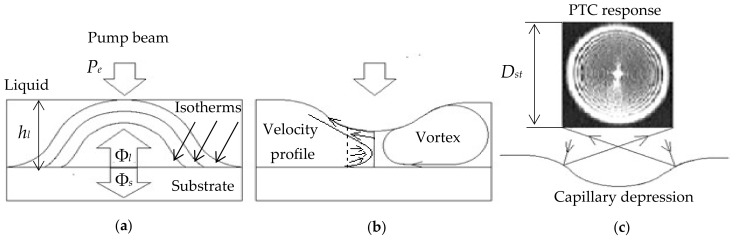
The photothermocapillary (PTC) effect: (**a**) isotherms and heat fluxes in a liquid; (**b**) urface deformation, formation of vortex and capillary depression; (**c**) PTC response.

Let us briefly describe the mechanism of formation of surface deformation in transparent liquids used in this study. Assume that there is a horizontal flat surface of a solid material (substrate) on which a liquid layer is deposited, whereby the liquid layer thickness $h_{l}$ is much less than linear dimensions of the substrate $d_{s}$, i.e., $h_{l}/\;d_{s}\ll 1$ (Figure 1a). The liquid layer is locally heated by a laser beam of the power $P_{e}$ absorbed by the substrate. During the heating of the substrate, a quasi-two-dimensional heat source is formed on its surface, and the thermal energy partially propagates into both the liquid (***Φ**_l_* is the conductive-convective heat flux) and substrate (***Φ**_s_* is the conductive heat flux), as shown in Figure 1a.

**Figure 2:**
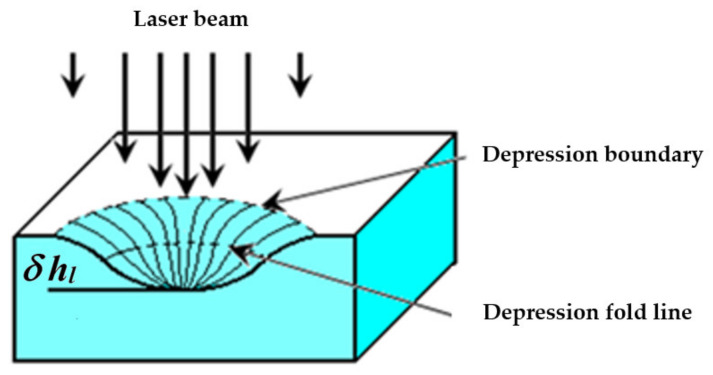
Cross-sectional view of the TC depression in a liquid layer.

There are isotherms accompanying the substrate heating and propagating from the liquid-substrate interface into the two media. The velocity of the isotherm propagation depends on the liquid/substrate thermal diffusivities $k_{l}$ and $k_{s}$. It can be assumed that, after the delay time $\tau_{d}$, the so-called “launching” isotherm reaches the surface of the liquid layer to cause a local change of temperature. The delay time $\tau_{d}=h_{l}^{2}/4k_{l}$ is determined at the level 1-$e^{-n}$ of the substrate temperature T as $\tau_{d}=h_{l}^{2}/4nk_{l},$ where n is a positive number [27]. Since the surface tension of most liquids decreases with increasing temperature, the $\sigma_{l}<\sigma_{s}$ condition takes place at the point of heating, and the surface Marangoni forces appear to pull the liquid from the heating region to the periphery, where $T=T_{0}$. Respectively, the tangential gradient of the surface tension equal to $\sigma^{\prime }\Delta T$ appears on the surface of the liquid. Due to the liquid viscosity μ, the liquid surface flow is transferred to the underlying and thus less heated layers, leading to liquid entrainment from the heating zone. This causes a local reduction of the liquid layer thickness $h_{l}$ and shapes a TC depression in the form of a concave mirror. A negative pressure arises in the layer under the TC depression, causing a reverse liquid flow. As a result, the liquid flows along a closed line, producing a toroidal TC vortex. It passes through the center of the depression, being symmetric in relation to the axis and perpendicular to the interface of liquid-substrate, as shown in Figure 1b. As the layer of the liquid warms up, the velocity of the liquid in the vortex, and, hence, the depth of the TC depression, increases to a certain stationary value. The test laser beam reflected from the TC depression is projected on the remote screen as an interference pattern of concentric rings with a wider and brighter outer ring. The quasi-stationary diameter $D_{st}$ of this ring is called the PTC signal, as shown in Figure 1c. In fact, the PTC effect can be considered as a particular stage of a more general concentration-capillary effect, which was discovered by Bezuglyi in 1975 while irradiating the iodine solution with a mercury lamp [28]. In its classical form, the TC phenomenon was studied in the late 1970s while irradiating heavy oil fractions with a Helium-Neon (He-Ne) laser [29,30]. The PTC signal is called both the diffraction [28] and interference [30] patterns. Note that, on the surface of the liquid in the TC flow, there is a balance of shear and viscous stresses:

(1) $$\mu \frac{\partial v_{r}}{\partial z}=\frac{\partial \sigma }{\partial r}=\frac{\partial \sigma }{\partial T}\frac{\partial T}{\partial r},$$

where z and r are the vertical and radial coordinates (Figure 1b), respectively. The hydrodynamic properties of a TC vortex were thoroughly studied by the authors of [31] in the case of the localized electric heating of a transparent liquid layer. Figure 2 shows the cross-sectional view of a TC depression having the deflection depth of $\delta h_{l}$ and representing an optical element of two mirrors, namely the central concave and annular convex mirrors separated by the fold line.

The grid method, which is based on determining a type of deformation (concave or convex) by the grid projection image obtained by the reflection of the light beam from the liquid layer, can be considered one of the first qualitative techniques used to study the profiles of TC deformation of liquid-free surfaces [32]. The deformation was induced by heating the liquid layer from the bottom with an electron beam produced by an oscilloscope cathode ray tube. A liquid layer in a glass cuvette lying on a milk glass plate was heated with a vertically directed laser beam, thus inducing a TC depression. The semi-transparent mirror transmitted the radiation of a flash tube located under the plate. The flash tube radiation was expanded by the collector. The thickness of the liquid layer was preliminarily calibrated using an optical gradient wedge. When the interference method was used [32], the information on the profile of the TC depression was obtained by analyzing the interaction between the incident and reflected laser beams. To analyze the surface profiles of TC depression, Bezuglyi and Flyagin used the technique of aluminum powder tracer particles having the diameter of 10–15 µm [31]. The PTC effect was induced from the bottom by heating the liquid with a thermocouple built in the flush with the surface of the ebonite substrate. The powder was poured into the area of the TC vortex illuminated by a laser knife. In this way, one can observe glowing particles through the flat wall made of glass. Based on the location data of real and imaginary images of particles, the positions of points at the “liquid-air” interface were determined. Hereinafter, we introduce the inspection technique based on the PTC effect.

An expanded low-power test laser radiation, which was applied to the TC depression at a small angle from the normal angle, reflects off the free surface of the liquid layer to produce a PTC interference pattern on a remote screen. A relative signal change, with the respect to the variation of a parameter to be analyzed, characterizes the sensitivity of the PTC technique. In the above-described case, the free surface of the thin liquid layer serves as a sensor to detect temperature variations, and the signal is the image of the rings on the screen, which can easily be evaluated by its size or converted into an electrical signal using a photodetector and a pinhole. A liquid layer lying on a solid substrate is characterized by the sensitivity, which is determined by its ability to respond to the deformation appearing on the liquid-free surface due to the heat transfer from a two-dimensional source operating at the “substrate-liquid” interface. It is assumed that a test beam does not affect the temperature at the heating point. This temperature depends on the pump laser power $P_{e}$, liquid properties (absorption coefficient $a_{l}$, surface tension σ and its temperature derivative σ′, thermal conductivity $K_{l}$ and viscosity μ), liquid layer thickness $h_{l}$, as well as substrate thermal properties ($K_{s}$ and $k_{s}$) and dimensions (diameter $d_{s}$ and thickness $h_{s}$). The thermal properties of both the liquid and substrate material affect the dimensionless temperature variations $\Theta_{i}$ at the “air-liquid” interface. The corresponding equation was proposed in the following form [33]:

(2) $$\Theta_{i}={\left[1+Bi\left(H+\frac{d_{s}/K_{s}}{h_{l}/K_{l}}\right)\right]}^{-1},\;$$

where $Bi=\alpha h_{l}/K_{l}$ is the Biot number; α is the heat exchange coefficient, Wm^−2^K^−1^); $H=h_{l}/h_{0}$ is the dimensionless thickness of the liquid layer ($h_{0}$ is the initial liquid layer thickness); $d_{s}$ and $h_{l}$ are the thicknesses of the substrate and liquid, respectively; and $K_{s}$ and $K_{l}$ are the thermal conductivities of substrate material and liquid, respectively.

Any sensor can be characterized by a sensitivity threshold, which is defined as the minimum detectable signal. The PTS signals reflect changes in the profile of the free surface of the liquid layer. Therefore, the magnitude of such signals, for example, the abovementioned quasi-stationary diameter $D_{st}$, depends on the angle of inclination of the tangent to the TC depression fold line, as shown in Figure 2. In the absence of TC deformation of the liquid layer, the test laser beam is reflected from the layer as from a flat mirror, being projected on the screen in the form of a Gaussian spot with the diameter $D_{0}$. Such signal can be obtained if at least one of the following conditions is valid: (1) $P_{e}$= 0; (2) liquid thickness $h_{l}\geq \sqrt{2\sigma /\rho g}$ (ρ is the density of the liquid, g is the gravitational acceleration); (3) $K_{l}/K_{s}\ll 1$; (4) the liquid viscosity μ is high. As mentioned above, a $D_{st}$ value is determined by the diameter of the outer wider and a brighter ring of the interference circular pattern. This parameter is generally evaluated in the PTC method. The parameters of circular interference patterns depend on the following factors: (a) the laser power $P_{e}$ and laser beam diameter $2w_{e}$ at the incidence point; (b) the temperature of the liquid T; (c) the thickness of the liquid $h_{l}$; (d) the presence of meniscus borders, which deform the liquid surface; (e) the thermal and optical properties of the substrate, such as the thermal conductivity $K_{s}$, specific heat $c_{s}$, and absorption coefficient $a_{s}$; (f) the thermal, rheological, dynamic, and optical properties of the liquid, such as the thermal conductivity $K_{l}$, specific heat $c_{l}$, viscosity μ, surface tension coefficient σ, thermal coefficients of volumetric expansion ∂β/∂T, and surface tension ∂σ/∂T, and absorption coefficient $a_{l}$. In addition, the diameter of the optical pattern depends on the distance between the liquid layer and the screen.

High sensitivity of the stationary PTC response toward variations in the thermal conductivity of solids is the basis for the development of a PTC technique to be used in the NDT of solid non-porous materials, for example, analyzing the material thermal conductivity and detecting subsurface defects, as well as thin coatings, for example, evaluating VPC thickness and detecting delaminations between a coating and a metal. Such technique involves the PTC effect, which is based on the fact that surface tension of a thin (~1 mm or less) plane-parallel layer of a liquid, such as polymethylsiloxane-based silicon oil, decreases if the surface temperature increases, for example, by absorbing the radiation of a pump laser. With the decreasing surface tension, the surface of the liquid bends with the formation of a depression, which can be recorded by means of a test laser. As a result of the interference, the beam of the test laser reflected from the depression produces the PTC pattern on the screen. The method is based on plotting the dependence of the pattern diameter $D_{st}$ on the thermal conductivity $K_{s}$ of solid materials, where the dependence of $D_{st}$ on the position of focused and unfocused pump laser beam can be used to determine the material thermal conductivity. The size of the subsurface defects can be evaluated by determining the $D_{st}$ values for the focused and unfocused pump laser beam.

### 2.2. Experimental Setup

**Figure 3:**
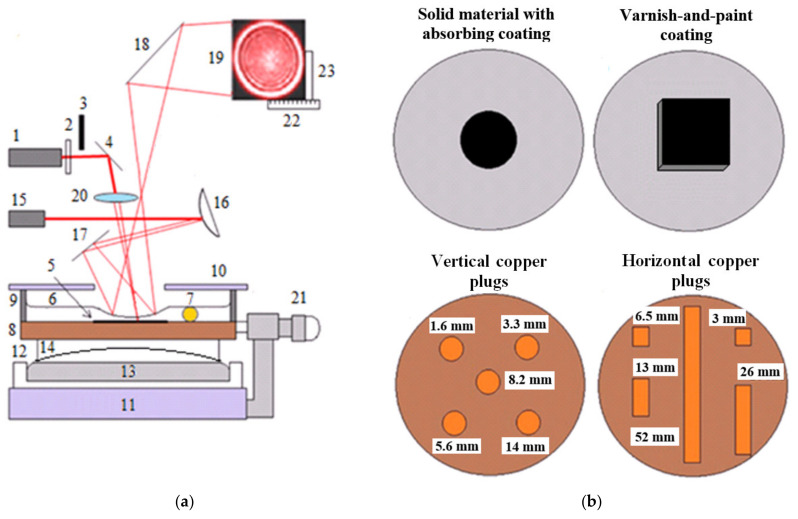
PTC setup (**a**) and top view of the sample under investigation (**b**): 1–pump laser, 2–light filter, 3–shutter, 4–mirror, 5–surface absorbing layer, 6–liquid layer, 7–wire, 8–solid material, 9–ring, 10–cover, 11–base, 12–Teflon cuvette, 13–Wood’s alloy, 14–plane-concave surface, 15–test laser, 16–18–mirrors, 19–screen, 20–lens, 21–microscrew, 22–ruler, 23–benchmark.

The experiments were carried out using the setup shown in Figure 3a. The beam of the pump laser 1 (He-Ne laser GN-25-1, maximum power $P_{e}$ = 21 mW, radiation wavelength λ = 632.8 nm, beam diameter $2w_{e}$ = 2.5 mm), was passed through the filter 2 and open shutter 3, and reflected from the mirror 4, and fell at a small angle from the normal angle on surface absorbing area 5, under the layer of oil 6. The oil layer thickness was set by a calibrated wire 7 of the known diameter and length (not more than 5 mm). The cuvette base 8 was a sample of a solid material, with the stopper ring 9 having the diameter of 50–60 mm and the height of 10 mm, and being fixed with epoxy adhesive. The liquid was protected from dust with the cover 10. The Teflon cuvette 12, with the hardened Wood alloy 13, was placed on the massive support base 11. The flat-concave lens 14 was accommodated on the surface of the Wood alloy and served as a subject table to move a sample. The expanded test laser beam 15 (He-Ne laser GN-3-1, power $P_{t}$ < 0.3 mW, λ = 632.8 nm, beam diameter $2w_{t}$ > 5 mm), after being reflected from mirrors 16 and 17, fell onto the TC depression of the liquid layer. The beam reflected from the depression and mirror 18 fell onto the remote screen 19 after travelling 3 m from the layer. The scanning was performed with the beam focused by the lens 20 with the focal length of 30 mm. In the experiments with the unfocused pump laser beam, the lens was replaced with a compensating glass plate. By rotating the microscrew 21 attached to the supporting base 11, the cuvette 7 was moved over the surface 14, and the stationary diameter $D_{st}$ of the PTC response was measured with the ruler 22. The edges of the response were marked with the benchmark 23 while searching for defect centers.

### 2.3. Test Samples

The next section presents the results of the application of the PTC method for he NDT of thin VPCs, particularly the evaluation of coating thickness variations and delaminations between a coating and a metal substrate [34,35,36,37,38].

The influence of the material thermal properties on the PTC response was investigated on the standard samples made of solid materials with thermal conductivities $K_{s}$ from 0.13 to 117 Wm^−1^K^−1^. Since the optical properties of the materials were different, to provide a high and uniform absorption of the pump laser radiation, a thin layer of Edding T25 ink was applied onto the samples polished with an abrasive paper of medium grain size (50 μm). Thus, after the evaporation of the solvent, a coating with the thickness of 10 μm was formed (Figure 3b). The liquid layer was produced by depositing PMS-5 oil.

To analyze the relationship between the PTC pattern diameter and the coating thickness, a film of a known thickness $h_{c}$ was adhesively attached to the surface of the duralumin base of the cuvette with zapon lacquer. Then, the PMS-5 oil was poured to produce a required oil film thickness controlled by the calibration wire.

The PTC method was also used to study the simulated defects in the solid materials and thin protective coatings. Air-filled voids and single vertical plugs were placed in the center of the cuvette base and covered with the VPC, which had a thickness of 45 ± 5 μm and a size of less than 1 × 1 cm (Figure 3b). In addition, the matrices of the vertical and horizontal plugs were placed over the entire surface of the cuvette base and perfused with zapon lacquer. The void-like defects were modeled, with holes having the diameter $d_{air}$ = 0.45 mm and height $h_{air}$ = 3 mm. Single plugs with the diameter of $d_{pl}$ = 2.2 mm and length of $l_{pl}=$ 14 mm were made of copper and ebonite, and adhesively inserted into ebonite and duralumin disks, respectively (Figure 3b). The vertical copper plugs was characterized by the same diameter of $d_{pl}$ = 2.2 mm, and varying lengths of $l_{pl}$ = 1.6, 3.3, 5.6, 8.2, and 14 mm were adhesively inserted into the ebonite disk (Figure 3b). The horizontal copper plugs ($d_{pl}$ = 2.2 mm, $l_{pl}$ = 3, 6.5, 13, 26, and 52 mm) were inserted into adhesive-filled grooves manufactured in the ebonite disk at a depth of 2.2 mm (Figure 3b).

## 3. Results

### 3.1. Material Thermal Conductivity and PTC Pattern Diameter

**Figure 4:**
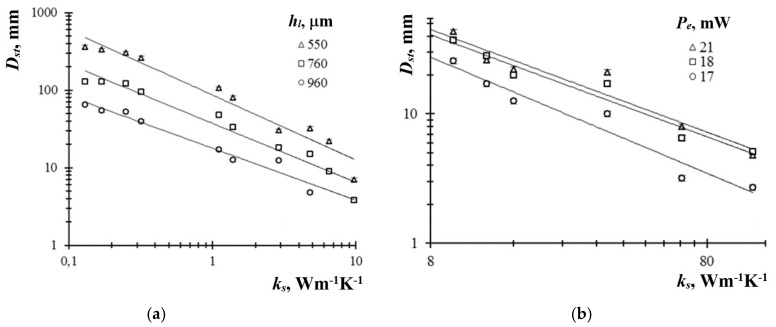
Dependences of the diameter $D_{st}$ of the PTC response on the thermal conductivity $K_{s}$: (**a**) low-conductive materials; (**b**) highconductive materials.

By analyzing the relationship between the material thermal conductivity and the PTC pattern diameter, it was found that the dependence $D_{st}\left(K_{s}\right)$ can be expressed by the function $K_{s}^{-\psi }$, which is logarithmically plotted in Figure 4. The index ψ increased from 0.67 to 0.84 with the decreasing thickness $h_{l}$ (at $P_{e}$ = 21 mW), and increased from 0.79 to 0.89 with the decreasing power $P_{e}$ (at $h_{l}$ = 100 µm). In the $K_{s}$ range from 0.13 to 0.17 Wm^−1^K^−1^, the dependences $D_{st}$$\left(K_{s}\right)$ and $D_{st}(h_{l})$ were as follows: $\Delta D_{st}/\Delta K_{s}$ = 59 mm/(Wm^−1^K^−1^) and $\Delta D_{st}/\Delta h_{l}$ = −0.72 mm/μm. For $K_{s}$= 6.5–9.7 Wm^−1^K^−1^, the corresponding relationships were $\Delta D_{st}/\Delta K_{s}$ = 0.47 mm/(Wm^−1^K^−1^) and $\Delta D_{st}/\Delta h_{l}$ = −0.05 mm/µm. When determining the $D_{st}$ with the error of 1 mm in the range of $K_{s}\;\leq$ 9.7 Wm^−1^K^−1^, the equivalent changes in thermal conductivity $\Delta K_{s}$ were 0.02–0.07 Wm^−1^K^−1^ for $h_{l}=$ 550–960 µm. For $K_{s}\geq$ Wm^−1^K^−1^, the limiting changes of $K_{s}$ were in the range of 0.2-0.4 Wm^−1^K^−1^ for $P_{e}$ = 17–21 mW. In the high-conductive solids, the decrease in the $D_{st}$ variations vs. thermal conductivity $K_{s}$ was due to the balance of heat fluxes, namely the diameter $D_{st}$ was determined by the fraction of the heat flux $\Phi_{l}$ propagating into the liquid, and the $\Phi_{l}$ was smaller with greater $K_{s}$. However, a reasonably high sensitivity of $D_{st}$ vs. $K_{s}$ for many solid materials is the basis for the development of methods to evaluate defects in coatings and solids.

### 3.2. Evaluating VPC Thickness

**Figure 5:**
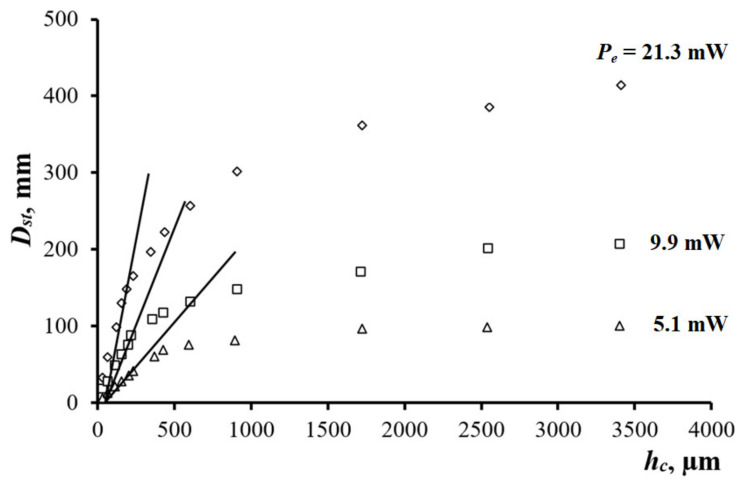
$D_{st}$ vs. $h_{c}$ (VPC on duralumin).

Evaluation of VPC thickness requires obtaining the calibration dependences of the response $D_{st}$ on the coating thickness $h_{c}$. The dependence $D_{st}\left(h_{c}\right)$ was plotted for several values of power $P_{e}$, as shown in Figure 5. In the $h_{c}$ range from 30 to 250 μm for a given thickness $h_{l}$ = 550 μm, the diameter $D_{st}$ grew linearly and reached saturation at $h_{c}$ > 4000 μm, indicating the presence of a certain threshold thickness. Such threshold corresponds to a thermally thick material and determines the work section of the $D_{st}\left(h_{c}\right)$ relationship, which is convenient for implementing the PTC inspection of film thickness. Increasing the power $P_{e}$ by a factor of four made possible to enhance the $D_{st}$ sensitivity vs. variations in coating thickness, expressed as $\Delta D_{st}/\Delta h_{c}$, from 0.21 ± 0.02 to 0.79 ± 0.17 mm/μm with the relative measurement error of about 5% (see the straight lines in Figure 5). Such error provides the determination of the VPC thickness with accuracy better than 10 μm, which is acceptable for many practical applications.

### 3.3. PTC Detection of Defects in Solids

**Figure 6:**
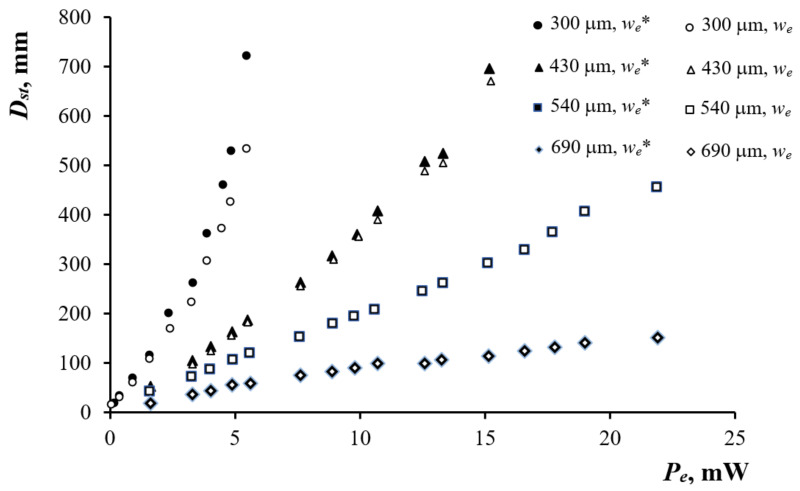
$D_{st}$ vs. $P_{e}$ (VPC on duralumin, PMS-5 oil layer on ebonite, focused and unfocused pump laser beam with $2w_{e}$ = 2.5 mm and $2w_{e}$* = 0.5 mm, superscript “*” defines focused laser beam).

The application of the PTC method as a technique of the NDT was studied on the test samples, as described in p. 2.3. The influence of the pump laser diameter $2w_{e}$ on the $D_{st}$ vs. $P_{e}$ dependence was analyzed in experiments where the PTC effect was induced in the PMS-5 oil layer on ebonite by both unfocused ($2w_{e}$ = 2.5 mm) and focused ($2w_{e}$* = 0.5 mm) pump laser beams. The diameter of the pump laser beam was estimated by the holes in the duralumin foil located between the lens and the head of the radiation power gauge. The hole diameters ranged from 0.5 to 3.0 mm with the step of 0.5 mm. The focusing of the pump laser beam increased the $\Delta D_{st}/\Delta P_{e}$ values from 156.7 ± 69.2 to 285.1 ± 90.2 mm/mW for the layer thickness of $h_{l}$ = 300 μm, as shown in Figure 6.

**Figure 7:**
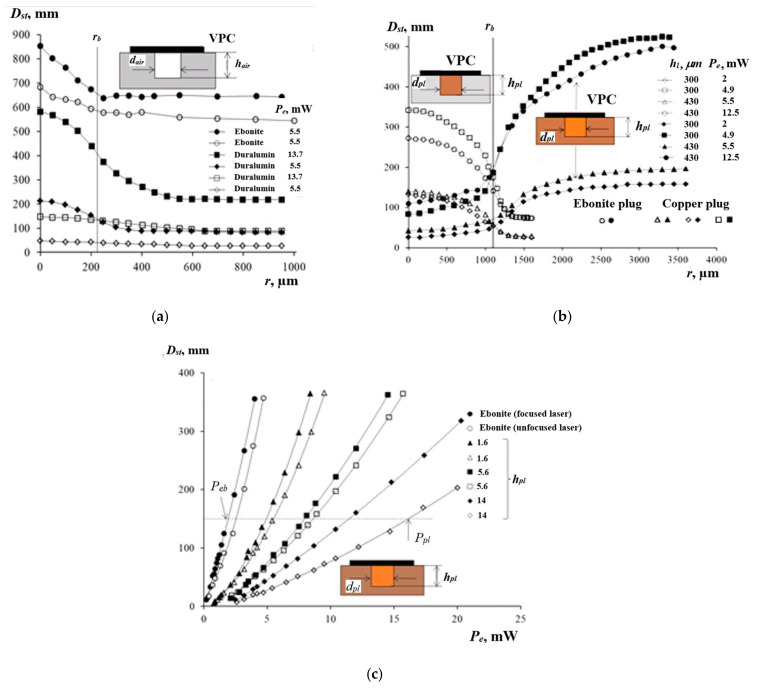
Evaluating the vertically oriented plug-like defects under the VPC ($h_{pl}$, $d_{pl}$–plug height and diameter): (**a**) scanned profiles over voids in ebonite/duralumin; (**b**) scanned profiles over copper and ebonite plugs; (**c**) $D_{st}$ vs. $P_{e}$.

Therefore, using a focused pump laser beam to induce the PTC phenomenon in a thin layer of a low-viscosity liquid allowed us to improve the resolution of the technique when detecting defects in solid materials. In the experiments on the air-filled voids, by moving the cuvette, the centers of the voids were evaluated by the maximal observed diameters $D_{st}$, determined by means of the benchmark. Afterward, the PTC patterns were scanned with the step of 50 μm to produce the scan profiles $D_{st}\left(r\right)$, as shown in Figure 7a. The corresponding void boundaries $r_{b}$ were determined by plotting the tangent to the curve $D_{st}\left(r\right)$ or using the half-amplitude method $A/2=\left(D_{max}-D_{min}\right)/2$), where $D_{max}$ and $D_{min}$ are the maximum (above the center of the void) and minimum (at the periphery) diameters of the response on the scan profile, respectively. The procedure was based on setting the second derivative of the approximating Gaussian function to zero, i.e., $D_{st}^{\prime \prime }\left(r\right)=0$, and using the finite difference equation $\left(D_{i+2}-2D_{i}+D_{i-2}\right)/{\left(\Delta r\right)}^{2}=0$. The half-amplitude method was proven to be simple and robust. In the case of the void in duralumin, the three-fold increase in the amplitude A, due to the application of a higher pump beam power, caused the error in determining the void boundary $r_{b}$ to drop from 7 to 2.7% if $2w_{e}$* = 0.5 mm. However, in the case of $2w_{e}$ = 2.5 mm, the same increase in the amplitude A reduced the error from 60 to 33%.

Figure 7b shows the scanned $D_{st}$ profiles obtained on single plugs made of copper and ebonite, and adhesively inserted into ebonite and duralumin disks, respectively. Since ebonite is less conductive than copper, the $D_{st}$ value at the periphery of the copper plug in ebonite was higher than over the plug center, and the reverse situation occurred over the ebonite plug in duralumin. In the case of the ebonite plug, the increase in amplitude A by 2.4 times diminished the error in determining the boundary $r_{b}$ from 18.4 to 6.3%. When evaluating the copper plug, the corresponding error of determining $r_{b}$ decreased from 30 to 22% with a 2.8-factor increase in the amplitude A.

When heating the centers of the plug ends’ faces, the $D_{st}$ vs. $P_{e}$ relationship was evaluated on the vertically oriented plugs with the laser beam diameters of $2w_{e}$ = 2.5 mm and $2w_{e}$* = 0.5 mm (layer thickness $h_{l}$ = 300 μm), as shown in Figure 7c. With the increasing length $l_{pl}$, the values of $\Delta D_{st}/\Delta P_{e}$ decreased by a factor of 4.5 (from 63 to 14 mm/mW) because of the increased heat diffusion into the solid material. The influence of the pump beam diameter $2w_{e}$ on $\Delta D_{st}/\Delta P_{e}$ was noticeable for the long plugs. With greater $l_{pl}$, the power loss $\Delta P_{e}=P_{pl}-P_{eb}$ increased, where $P_{pl}$ and $P_{eb}$ are the powers of the laser beam, which are necessary to produce the same values of $D_{st}$ in the absence and presence of plugs, as shown by the horizontal dashed line in Figure 7c. For a focused beam, the saturation of the function $\Delta P_{e}\left(l_{pl}\right)$ occurred earlier than the unfocused beam, indicating limitations in using focused beams for the diagnostics of plugs in a wide range of $l_{pl}$. The sensitivity of the method for the vertical plugs was $\Delta D_{st}/\Delta l_{pl}$ = −112 (for $l_{pl}$ = 0–1.6 mm).

**Figure 8:**
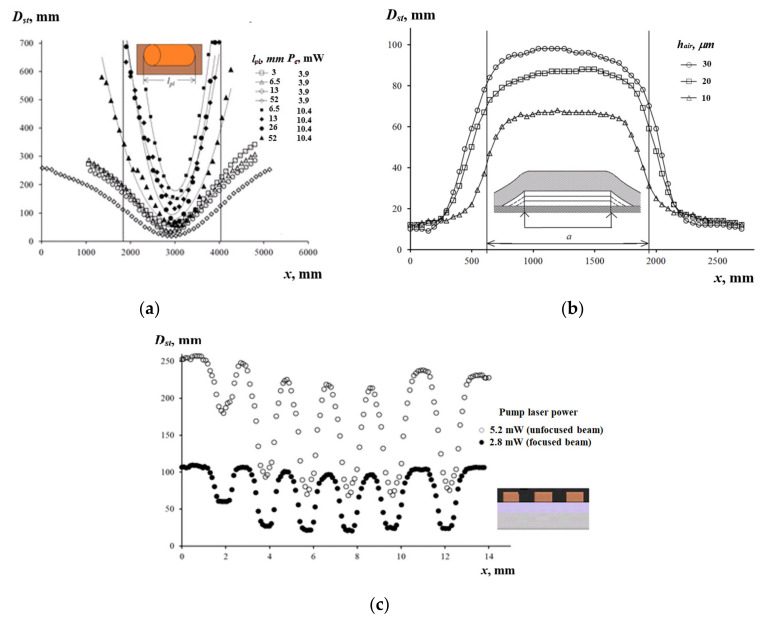
Evaluating the horizontally oriented defects: (**a**) matrix of plugs; (**b**) coating delamination (a is the delamination width); (**c**) copper paths of printed circuit board (PCB).

The scanning of the matrix of the plugs allowed the analysis of the influence of plug height and length on the $D_{st}$ with a single cuvette. The horizontal plugs were scanned with the 100 μm step perpendicularly to the line, which passed through their midpoints, to produce scan profiles $D_{st}\left(x\right)$ by the coordinate x (Figure 8a). With the increasing $l_{pl}$, the values of $\Delta D_{st}/\Delta x$ at the defect boundary decreased more quickly for the focused beam ($2w_{e}$* = 0.5 mm) compared to the unfocused beam ($2w_{e}$ = 2.5 mm). For the plug length of $l_{pl}$ = 52 mm, the increase in the power $P_{e}$ from 3.9 to 10.4 mW caused the $\Delta D_{st}/\Delta x$ values to increase by nine times, while the $D_{st}$ values over the plug centers increased by three times.

Delaminations between the VPC and the copper sample were simulated by a rectangular air void with stepped channels by inserting and removing a Teflon tape, which was 1.4 mm wide and 10 μm thick (Figure 8b). The air gap thicknesses were $h_{air}$ = 10, 20, and 30 μm. The delamination was scanned by the laser beam ($P_{e}$ = 4.6 mW, $2w_{e}$* = 0.5 mm) with the step of 50 μm along the line perpendicular to the delamination. The error in evaluating the $D_{st}$ was 3% in the center of the delamination and increased to 13% at the border. The scan profile width increased with greater $h_{air}$ because of a higher thermal resistance of the wedge-shaped gap. In the range of $h_{air}$ from 10 to 30 µm, the diameter $D_{st}$ of the response over the delamination centers grew linearly and saturated at $h_{air}=$ 130 µm. The $D_{st}\;vs.\;h_{air}$ dependence was obtained for three values of the VPC delamination and approximated by the function $D_{st}\left(h_{air}\right)=D_{uc}+(D_{uc}-D_{\infty })\;\left[1-Exp\left(-0.0463\;h_{air}\right)\right]$, where the difference $D_{uc}-D_{c}$ was determined by peeling off the VPC, and $D_{uc}=D_{st}\left(h_{air}=0\right)$ represented the start point for calculating $h_{air}$. The diameter $D_{uc}$ characterized the size of the optical pattern produced by a VPC film, which was placed on a metallic substrate. It was determined as a cross-section point of the $D_{st}\left(h_{air}\right)$ dependence with the vertical $D_{st}$ axis, while the diameter $D_{c}$ was found experimentally when exciting the PTC effect at the point with no delamination. The limiting value $D_{\infty }=D\left(h_{\infty }\right)$ was measured as the response produced by the hole (diameter 1.4 mm, depth 2 mm) located in duralumin under the VPC film. The experiment, which was performed on holes located in a copper substrate under the VPC of the same thickness, resulted in the same value $D_{st}$=$D_{\infty }\left(h_{air}=500\;\mu m\right)=$ 135 mm. Therefore, we accepted that $h_{air}=$ 2 mm knowingly meets the $h_{air}\geq h_{\infty }$ condition. In this way, the error in determining the delamination boundaries did not exceed 11%. The sensitivity of the method for small $h_{air}$ was $\Delta D_{st}/\Delta h_{air}=$ 2.1 ± 0.8 mm/μm. The corresponding absolute error in determining the air gap thickness was evaluated to be under 1 μm for $h_{air}$ =10 μm, and the maximum value of $D_{st}$ = 65 mm provided the accuracy of evaluating $D_{st}$ of about 5%.

The PCB copper paths, which are 2, 4, 8, 16, 32, and 64 mm long; 1.4 mm wide; and separated by 1.3 mm-wide insulating gaps, were etched in a ferric chloride solution. Then, the PCB was attached with epoxy adhesive to the duralumin disk, which acted as a heat sink and stiffener (Figure 8c). The paths were scanned at $P_{e}$ = 2.8 and 5.2 mW, with the step of 100 μm along the line connecting their midpoints perpendicularly to the path axes (Figure 8b). The scan profile included a sequence of the maximum $D_{max}$ (the beam was between two adjacent paths) and minimum $D_{min}$ (the beam was over a path center) values of the response diameter. Both values diminished with a greater path length. The latter phenomenon can be explained by a very high thermal conductivity of copper. The measurement sensitivity $\Delta D_{min}/\Delta l$ was −16 and −43 (in dimensionless units), and the error of diameter measurement was $\Delta D_{min}$ = ± 1 mm, allowing the detection of variations in the length l of the copper tracks equal to Δl = 23 and 62 µm. Note that the critical values of the length $l_{c}$, which corresponded to saturation of the $D_{min}\left(l\right)$ function, were 4.6 and 5.0 mm for $P_{e}$ = 2.8 and 5.2 mW, respectively.

In the PTC technique described above, the main sources of $D_{st}$ measurement errors are: (1) a level of PTC screen luminance that influences visibility of optical patterns; (2) variations in the VPC thickness and defect size, which set a task of defect characterization; and (3) random deviations of the temperature profiles from the defect centers, affecting the results of the defect size evaluation, and leading to difficulty in defining defect centers. Additional measurement errors are provided by: (1) variations in the liquid layer thickness because of its wedge shape and surface roughness, and (2) weak fluctuations of the pump laser power. A decent evaluation of PTC measurement errors requires further analysis. In addition, it is worth mentioning that the NDT applications of the PTC method are mainly related to defect detection rather than defect characterization.

## 4. Discussion

The PTC technique was developed to monitor the thickness of VPCs ($h_{c}$) on thermally conductive solid materials by measuring a specific PTC parameter $D_{st}$. A four-fold increase in the test laser power $P_{e}$ enhanced the sensitivity $\Delta D_{st}/\Delta h_{c}$ from 0.21 ± 0.02 to 0.79 ± 0.17 mm/μm with a relative $D_{st}$ measurement error of 5% (for $h_{c}$ = 30–250 μm).

The same PTC technique was used to monitor the thickness $h_{air}$ of air-filled delaminations between metals and VPCs having a coating thickness from 10 to 30 μm. The PTC pattern diameter was linearly proportional to the air gap thickness. The error in determining the defect boundaries was under 11%, and the sensitivity of the method was $\Delta D_{st}/\Delta h_{air}$ = 2.1 ± 0.8 mm/μm for small $h_{air}$. A decrease in the laser beam diameter and/or an increase in the laser power led to an increase in the inspection sensitivity $\Delta D_{st}/\Delta x$.

The above method was used to estimate the length $l_{pl}$ of the vertical and horizontal copper plugs by the parameter $D_{st}$. The sensitivity achieved by measuring the vertical plugs and horizontal plugs was $\Delta D_{st}/\Delta l_{pl}$ = −112 (for $l_{pl}$ = 0–1.6 mm) and $\Delta D_{st}/\Delta l_{pl}$ = −64 (for $l_{pl}$ = 0–3.3 mm) in dimensionless units, respectively.

It has also been demonstrated that the PTC technique can be used to evaluate the quality of conductive PCBs.

The resolution of the test method can be improved by diminishing the diameter of a pump laser beam to the values of $2w_{e}$* < 0.5 mm using thinner layers (thickness $h_{l}$ < 300 μm) of less viscous liquids (μ < 4.6 mPa·s). In general, we believe that the PTC analysis can be applied to convex samples to detect voids, solid inclusions, and delaminations in materials and VPCs. The method is expected to be efficient in detecting small (<0.5 mm) defects and inspecting thin (<30 μm) coatings.

Future research will focus on a deeper analysis of the measurement errors and their dependence on the diameter of a focused pump laser beam, as well as the determination of the acceptable ratio between the thermal conductivities of a material and defects.

## 5. Conclusions

This study establishes the main features of the PTC method for the detection of subsurface defects and the evaluation of coating thickness, as well as the determination of material thermal properties. The proposed NDT technique is applicable to materials and defects in a wide range of thermal conductivities. It seems to be promising for evaluating defects less than 1 mm in size and coatings with thicknesses over 10 µm. The linear dimensions of air-filled delaminations between a metal substrate and a coating, as well as other defects in solids, can be accurately determined using the half-amplitude technique.
